# Inferring social influence in animal groups across multiple timescales

**DOI:** 10.1098/rstb.2022.0062

**Published:** 2023-04-10

**Authors:** Vivek H. Sridhar, Jacob D. Davidson, Colin R. Twomey, Matthew M. G. Sosna, Máté Nagy, Iain D. Couzin

**Affiliations:** ^1^ Department of Biology, University of Konstanz, 78464 Konstanz, Germany; ^2^ Centre for the Advanced Study of Collective Behaviour, University of Konstanz, 78464 Konstanz, Germany; ^3^ Department of Collective Behaviour, Max Planck Institute of Animal Behavior, 78464 Konstanz, Germany; ^4^ Department for the Ecology of Animal Societies, Max Planck Institute of Animal Behavior, 78467 Konstanz, Germany; ^5^ Department of Biology, University of Pennsylvania, Philadelphia, PA 19104, USA; ^6^ Mind Center for Outreach, Research, and Education, University of Pennsylvania, Philadelphia, PA 19104, USA; ^7^ Department of Ecology and Evolutionary Biology, Princeton University, Princeton, NJ 08544, USA; ^8^ MTA-ELTE Statistical and Biological Physics Research Group, Hungarian Academy of Sciences, Budapest 1117, Hungary; ^9^ MTA-ELTE ‘Lendület’ Collective Behaviour Research Group, Hungarian Academy of Sciences, Eötvös Loránd University, Budapest 1117, Hungary; ^10^ Department of Biological Physics, Eötvös Loránd University, Pázmány Péter sétány 1A, Budapest 1117, Hungary

**Keywords:** leadership, collective behaviour, movement ecology, timescales, schooling, flocking

## Abstract

Many animal behaviours exhibit complex temporal dynamics, suggesting there are multiple timescales at which they should be studied. However, researchers often focus on behaviours that occur over relatively restricted temporal scales, typically ones that are more accessible to human observation. The situation becomes even more complex when considering multiple animals interacting, where behavioural coupling can introduce new timescales of importance. Here, we present a technique to study the time-varying nature of social influence in mobile animal groups across multiple temporal scales. As case studies, we analyse golden shiner fish and homing pigeons, which move in different media. By analysing pairwise interactions among individuals, we show that predictive power of the factors affecting social influence depends on the timescale of analysis. Over short timescales the relative position of a neighbour best predicts its influence and the distribution of influence across group members is relatively linear, with a small slope. At longer timescales, however, both relative position and kinematics are found to predict influence, and nonlinearity in the influence distribution increases, with a small number of individuals being disproportionately influential. Our results demonstrate that different interpretations of social influence arise from analysing behaviour at different timescales, highlighting the importance of considering its multiscale nature.

This article is part of a discussion meeting issue ‘Collective behaviour through time’.

## 1. Introduction

Spectacular displays of coordinated group movement are widespread across the animal kingdom—from fish schools [1,2] exhibiting near-instantaneous changes in their direction, to locust swarms [3,4] marching across the desert, to pigeon [5,–8] and starling [9–11] flocks weaving through the skies to escape predators. A hallmark of such collective response is that the movement—and especially changes in movement—of each individual has the potential to influence the behaviour of other group members, which in turn can trigger behavioural changes in other group members, and so on. A key aspect in understanding collective behaviour, therefore, is establishing the degree to which each individual has the capacity to influence others, often referred to in the literature as ‘leadership’ [8,12] or ‘social influence’ [13].

Within most animal groups influence changes dynamically over time, and is thus distributed among members of the group. All individuals, over a course of time, either directly or indirectly influence each other's movement to some degree. However, within-group heterogeneity in various traits has been found to give some individuals more influence over the group's movement direction than others. For example, body size [14], relative speed [15,16], navigational experience [17], sociability [15,18], motivation [18], social status [19], informational status [2] and spatial position [19–21] within the group have all been found to be correlated with social influence within groups. Driven by such a diverse range of mechanisms, collective behaviour inherently exhibits complex and multiscale temporal dynamics. Furthermore, when animals interact with one another they can mutually, and recursively, influence each others' behaviour. This itself can introduce further timescales not present when individuals are in isolation. Thus, although considerable progress has been made in recent years in the quantification of behaviour [15,22–26] and influence [13], there still exist many challenges in correctly identifying and interpreting the multiscale nature of both individual and collective animal behaviour [27].

**Figure 1.**
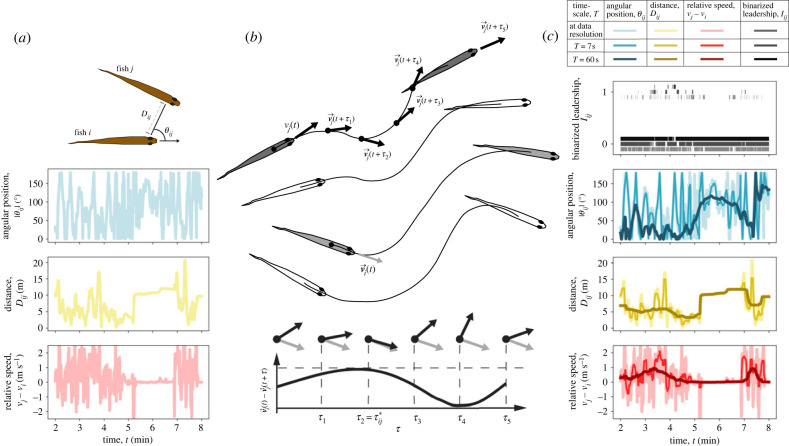
**Summary of the leader–follower analyses conducted over multiple timescales.** (*a*) Temporal dynamics of some features used to predict social influence in animal groups. The top panel is an illustration of angular position (*θ_ij_*) and metric distance (*D_ij_*) for two neighbouring fish. The following three plots in (*a*) show short time segments of angular position (in blue), distance (in yellow) and difference in movement speed of a pair of individuals (in red) at the resolution at which data were recorded. (*b*) A schematic of the time-lagged directional cross-correlation technique (adapted from [8]) used to analyse leader–follower relationships between pairs of individuals. For each pair (*i* ≠ *j*), the directional correlation function is $C_{ij}(t^{\prime })={\left\langle v_{i}(t^{\prime })\cdot v_{j}(t^{\prime }+\tau )\right\rangle }_{t^{\prime }\in [t-(T/2);\;t+(T/2)]}$, where 〈…〉 denotes a time average at the specified timescale. Below is a visualization of the dot product of the normalized velocity of individual *i* at time *t* and that of individual *j* at time *t* + *τ* (modified from [8]). (*c*) Illustration of the leadership scores over multiple timescales for the same sample period shown in (*a*) along with angular position, distance and relative speed at corresponding timescales.

Here, we analyse fine-scale collective movement of two very different model systems, schooling golden shiner fish (*Notemigonus crysoleucas*) and flocking homing pigeons (*Columba livia*), at multiple timescales. First, we obtain position and kinematic properties—speed and acceleration—of each individual relative to every other individual in the group (figure 1*a*). Second, we use the time-lagged directional cross-correlation technique [8,28] to analyse leader–follower relationships between pairs of individuals (figure 1*b*). While numerous other methods have previously been used to quantify social influence [13], we chose this approach as it is simple and also agnostic to the exact nature of the social interactions in which individuals engage. Third, we combine the above metrics at different timescales to explore the predictive power of relative position and kinematic properties in predicting leadership (relative social influence) in groups (figure 1*c*). Taken together, our work reveals that the strength of predictions made by the different variables (positional or kinematic) on leadership, and the inferred distribution of leadership across members of the group, can change considerably depending on the timescale of analysis.

## 2. Material and methods

### (a) Datasets

In this paper, we re-analysed two datasets from previous studies [29,30], which contained trajectories of freely swimming schooling golden shiners (*Notemigonus crysoleucas*) [1,31–33] extracted from video data [29], and GPS trajectories of freely flying pigeon (*Columba livia*) [21,34–38] flocks near their home lofts [30]. Below, we describe some of the details specific to each dataset:

**(i)** **Golden shiners.** Groups of 10 and 30 golden shiners (*Notemigonus crysoleucas*) were allowed to swim freely in a 2.1 × 1.2 m experimental tank containing a 4.5–5 cm depth of water. Fish were filmed at 30 Hz for 2 h using a Sony EX-1 camera placed 2 m above the tank. Three trials were conducted per group size and fish were not reused over multiple trails. Our analyses are focused on 13 min segments from each trial. Chosen segments started 1 h after the onset of the trial to minimize effects of stress on the fish from handling. Fish positions, orientations and body sizes were extracted from videos using SchoolTracker [1] and manual data correction was performed to ensure accuracy of the tracks and to maintain individual identities over time.
**(ii)** **Homing pigeons.** GPS data were collected at 10 Hz from free flights of flocks of homing pigeons (*Columba livia*) around the loft. All 30 pigeons were aged 2.8 ± 1.6 years (mean ± s.d.) and came from two neighbouring lofts at the University of Oxford Field Station. Birds were allocated to three groups of 10 (A, B and C) and were also tested in a combined group of 30 (group ABC). All analyses were conducted on two flights each for groups A, B and C, and one flight for group ABC. We analysed data in two dimensions (using *x*- and *y*-coordinates) on flight segments that ranged between 8 and 40 min in duration.

### (b) Data analyses

**(i)** **Processing trajectory data.** First, trajectories obtained from the two datasets were used to calculate kinematic properties, i.e. speed and acceleration, of individuals (see electronic supplementary material for the details on calculating these variables). Since our interest is to examine social influence at the dyadic level, we also computed pairwise features from the trajectories. At each frame, we determined the relative position of each individual with respect to every other individual in the group. This was measured as an egocentric vector-based representation of the neighbour i.e. the angular position and distance of a neighbour relative to a focal individual (figure 1*a*; see electronic supplementary material for the details on calculating positional variables). We also calculated relative kinematics, i.e. the difference in speed and acceleration between a focal individual and its neighbours (figure 1*a*; see electronic supplementary material for the details on calculating kinematic variables). Once all relevant features were computed for every individual (and pair) across the entire dataset, we used a moving window average to analyse our data at four different timescales—*T* ∈ {7, 15, 30, 60 s}. These were the timescales of our analysis.
**(ii)** **Calculating influence.** To calculate the influence of a given individual on its neighbour, we adopted the time-lagged directional correlation technique [8,12,28] with a moving time window—*T* ∈ {7, 15, 30, 60 s}. Note that we use the terms 'influence' and 'leadership' interchangeably; in doing so, we refer to this time-lagged directional correlation definition. At each time step *t*, we calculated the time-lagged correlations between directions of pairs of individuals *C_ij_*(*τ*, *t*),
  2.1 $$C_{ij}(\tau ,t)={\left\langle \frac{v_{i}(t^{\prime })}{\left\|v_{i}(t^{\prime })\right\|}\cdot \frac{v_{j}(t^{\prime }+\tau )}{\left\|v_{j}(t^{\prime }+\tau )\right\|}\right\rangle }_{t^{\prime }\in [t-(T/2);\;t+(T/2)]},$$
  where $v_{i}(t)$ denotes the velocity vector of individual *i* at time *t*, $v_{i}(t)/\|v_{i}(t)\|$ denotes the unit direction of individual *i* at time *t* and 〈…〉 denotes a time average at the specified timescale. For each time step *t*, the maximum value of the correlation function *C_ij_*(*τ*, *t*) was determined as $C_{ij}^{*}(t)\;$ and the corresponding time lag *τ_ij_*(*t*) was determined as $\tau_{ij}^{*}(t)$ (figure 1*b*). Positive $\tau_{ij}^{*}(t)$ values correspond to being followed—when the directional motion of the focal individual *i* is ‘copied’ by its neighbour *j*—while negative $\tau_{ij}^{*}(t)$ values correspond to following—when the focal individual *i* ‘copies’ directional motion of its neighbour *j*. To ensure that the correlation value holds meaningful information about directional copying, we only consider periods when the average directional difference (within the analysis timescale) is less than 30° i.e. periods when $C_{ij}^{*}(t)\geq \sqrt{3}/2$ as leading events. We also disregarded events where the time lag $|\tau_{ij}^{*}(t)|\leq 1/6\;s$ as we considered this time window to be too short for directional copying. As demonstrated by a sensitivity analysis, detailed in the electronic supplementary material, our results are insensitive to the exact values chosen for these two thresholds (angular threshold and time threshold; electronic supplementary material, figures S1–S4). Using these criteria, we simplified the cross-correlation values and classified interactions between all dyads across all frames ${\tilde{l}}_{ij}(t)$ as being engaged in a leader–follower relationship, or not:
  2.2 $${\tilde{l}}_{ij}(t)=\{\begin{array}{cc} C_{ij}^{*}(t) & \mathrm{if}\;\;C_{ij}^{*}(t)\geq \frac{\sqrt{3}}{2}\;\;\mathrm{and}\;\;|\tau_{ij}^{*}(t)|>1/6\;s\\ 0 & \;\mathrm{otherwise}. \end{array}$$
  Since in our study cases all individuals in a group are relatively well-aligned, we further filtered the above leadership scores and binarized them (figure 1*c*) such that, for a given individual *i*, only a single leader—the one with the largest correlation—is considered [39]:
  2.3 $$l_{ij}(t)=\{\begin{array}{rl} & 1\;\mathrm{if}\;{\tilde{l}}_{ij}=\max({\tilde{l}}_{ij}:j=1,2,\ldots ,N;\tau_{ij}^{*}<0;j\neq i)\\ & \;\;\mathrm{and}\;\;{\tilde{l}}_{ij}\neq 0\\ & 0\;\;\;\mathrm{otherwise}, \end{array}$$
  where *l_ij_*(*t*) is the binarized leadership score for dyad *i*,*j* at time *t* and *N* is the number of individuals in the group. The network of inter-individual interactions obtained from the binarized leadership score *l_ij_*(*t*) was then used to calculate the local reaching centrality [40] *R_k_*(*t*). This measure quantifies the proportion of the group influenced by individual *k* (both directly and indirectly) at time step *t*. By aggregating reaching centralities over time, we obtain influence scores *R_k_* for all individuals. An assessment of the distribution of influence across the group is then conducted by calculating the Gini coefficient of the individual influence scores (*R_k_*).
**(iii)** **Predicting influence across timescales.** To assess the strength of prediction of positional and kinematic properties on leadership (*l_ij_*) at each considered timescale, we constructed a balanced dataset—with an equal number of points where pairs of individuals exhibited a leader–follower relationship and where pairs did not exhibit this relationship—following which we fitted a Bayesian GLM to the data. The response variable was Bernoulli distributed and represented whether or not the pair exhibited a leader–follower relationship at that moment in time (*l_ij_*(*t*)). To explore the role of the different predictors, we constructed three different models denoted as M1, M2 and M3: M1, with relative position as a predictor—given by distance to, and angular position of, the neighbour relative to the focal individual; M2, with absolute and relative kinematics as predictors—speed, acceleration and difference in speed and acceleration between a focal individual and its neighbour; and M3, with both relative position and kinematics as predictors. Note that M3 combines all predictors used in the above models M1 and M2.
  For each model, we estimated the posterior *P*(*θ_μ_*|*X*, *y*) using the No-U-Turn Sampler (NUTS), a self-tuning variant of the Hamiltonian Monte Carlo (HMC) algorithm implemented in Stan (https://www.jstatsoft.org/article/view/v076i01). We drew HMC samples using four independent Markov chains consisting of 1000 warm-up iterations and 1000 sampling iterations, making a total of 4000 sampling iterations. To speed up sampling, we used a balanced dataset consisting of 5000 data points when fitting each generalized linear model. To balance the dataset, we randomly subsampled from our original dataset such that we had 2500 data points where pairs of individuals engaged in a leader–follower relationship, and 2500 data points where this was not true (see electronic supplementary material, tables S1 and S2 for details regarding the subsampling). Even though the proportion of data used in our GLMs is small (as shown in electronic supplementary material, tables S1 and S2), we ensured that it is representative of the entire dataset by assessing the model fit on five independent subsamples of the data. All models converged without any signs of pathological behaviour, and priors were chosen to be relatively uninformative distributions $\theta_{\mu }∼N(0,\;10)$.
**(iv)** **Temporal dynamics of social influence.** The analysis conducted above provides insights into factors that are correlated with leadership. However, to investigate the differences in the strength of prediction between the different timescales, we examined the temporal dynamics of leader–follower events by analysing the process as a time series. To this end, we isolated 40 s segments centred around 2 s where the leadership dynamics changed (pairs of fish exhibit a leader–follower relationship for 1 s followed by a 1 s period where they abandon this relationship, and *vice versa*). Averaging across all isolated time series segments, we asked: What features predict that an individual will start following a certain neighbour? or What features predict that an individual will stop following its current leader? Statistically, these two time series can be thought of as representing a switch in leadership from a focal follower's perspective. Finally, the isolated time series events were bootstrapped to obtain 95% confidence intervals around the means.

## 3. Results

We use two approaches to examine social influence among pairs of individuals in two case studies—swimming golden shiner schools and flying homing pigeon flocks. First, we use Bayesian GLMs to identify the relationship between position and kinematic properties of individuals relative to their neighbours, and their tendency to influence the movement direction of these neighbours. Based on their direct and indirect influence on group members, we get an influence metric for each individual in the group, measured here as the aggregate local reaching centrality (*R_k_*) of the individual. We subsequently quantify the distribution of influence across group members by calculating the Gini coefficient of this influence score (*R_k_*). All analyses are conducted at multiple temporal scales, and call attention to the timescale-dependent nature of our results. Second, we conduct a time series analysis of leader–follower events. In this analysis, the temporal sequence of relative positions and kinematics—before, during and after leadership—allow us to evaluate their association with leadership and inform us why we see timescale-dependence in the previous analysis. Below, we discuss results from both these analyses.

### (a) Timescale-dependence of social influence

**Figure 2.**
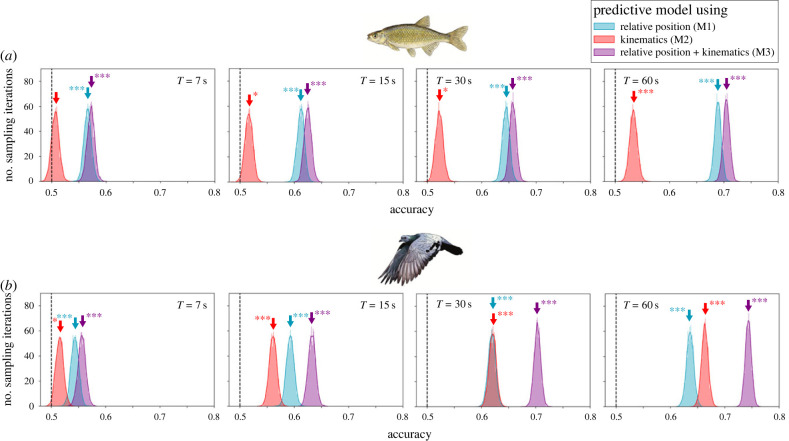
**Timescale-dependence of social influence.** Prediction accuracy of three models in a dataset of 5000 randomly selected data points for (*a*) golden shiners and (*b*) homing pigeons. The *y*-axis represents the number of sampling iterations (from a total of 4000) at any given accuracy. The four panels within each species represent four different timescales of analysis—*T* ∈ {7, 15, 30, 60 s}. Across models, and within the explored timescales, predictability of social influence grows as we analyse data over longer timescales. Coloured arrows represent the median accuracy of corresponding distributions and asterisks next to them indicate which distributions are significantly different from a chance expectation (shown here by the dashed line). The adopted criteria for significance are *p < 0.05, **p < 0.01 and ***p < 0.001.

The three different models that we constructed (M1, M2 and M3) to predict leadership based on relative position and kinematics of dyads performed differently across timescales and species. For both species, within the range of timescales analysed, we observe that the predictability of leadership increases when the data are analysed over longer timescales (figure 2; see tables 1 and 2 for prediction accuracies across timescales for golden shiners and homing pigeons respectively). At short timescales (*T* ≤ 15 s), relative position of the leader with respect to the follower (M1) is found to be a strong predictor of influence, with nearer individuals occupying relatively frontal positions being most influential. Contrary to our expectation [15,16], kinematic properties (M2) are found to have low predictive power (figure 2). At longer timescales (*T* ≥ 30 s), both relative position and kinematics predict leadership (figure 2). On the one hand, in golden shiners, despite an increase in predictive power of kinematics, we find that relative position is still the strongest predictor of social influence (at *T* = 60 s mean prediction accuracy 68.88% was higher for model M1 than 53.36% for model M2). On the other hand, in homing pigeons, a reversal in the strength of prediction made by relative position (M1) and kinematics (M2) is observed at longer timescales (median prediction accuracy 63.62% was lower for M1 as compared with 66.36% for M2). At these long timescales, kinematics become a better predictor of leadership than relative position (compare panels in figure 2*b*). For both species, across timescales, a combined model (M3) using both relative position and kinematics best predicts leadership (figure 2; see tables 1 and 2 for prediction accuracies).

**Table 1.** Prediction accuracies of GLMs for golden shiner fish: mean and 95% confidence intervals on prediction accuracy of the three models M1, M2 and M3 at the considered timescales *T* ∈ {7, 15, 30, 60 s}.

timescale	relative position (M1)			kinematics (M2)			relative position + kinematics (M3)		
	mean	2.5%	97.5%	mean	2.5%	97.5%	mean	2.5%	97.5%
*T* = 7 s	56.61	55.24	58.00	50.64	49.34	52.06	57.19	55.84	58.56
*T* = 15 s	61.15	59.80	62.46	51.66	50.26	53.02	62.48	61.18	63.82
*T* = 30 s	64.46	63.20	65.72	52.24	50.88	53.62	65.75	64.50	67.02
*T* = 60 s	68.88	67.68	70.04	53.36	51.94	54.72	70.51	69.36	71.68

**Table 2.** Prediction accuracies of GLMs for homing pigeons: mean and 95% confidence intervals on prediction accuracy of the three models M1, M2 and M3 at the considered timescales *T* ∈ {7, 15, 30, 60 s}.

timescale	relative position (M1)			kinematics (M2)			relative position + kinematics (M3)		
	mean	2.5%	97.5%	mean	2.5%	97.5%	mean	2.5%	97.5%
*T* = 7 s	54.48	53.12	55.86	51.48	50.12	52.88	55.72	54.34	57.18
*T* = 15 s	59.26	57.96	60.60	56.08	54.68	57.46	63.26	61.88	64.56
*T* = 30 s	61.90	60.56	63.18	62.04	60.68	63.40	70.30	69.06	71.48
*T* = 60 s	63.62	62.32	64.90	66.36	65.12	67.54	74.34	73.24	75.48

**Figure 3.**
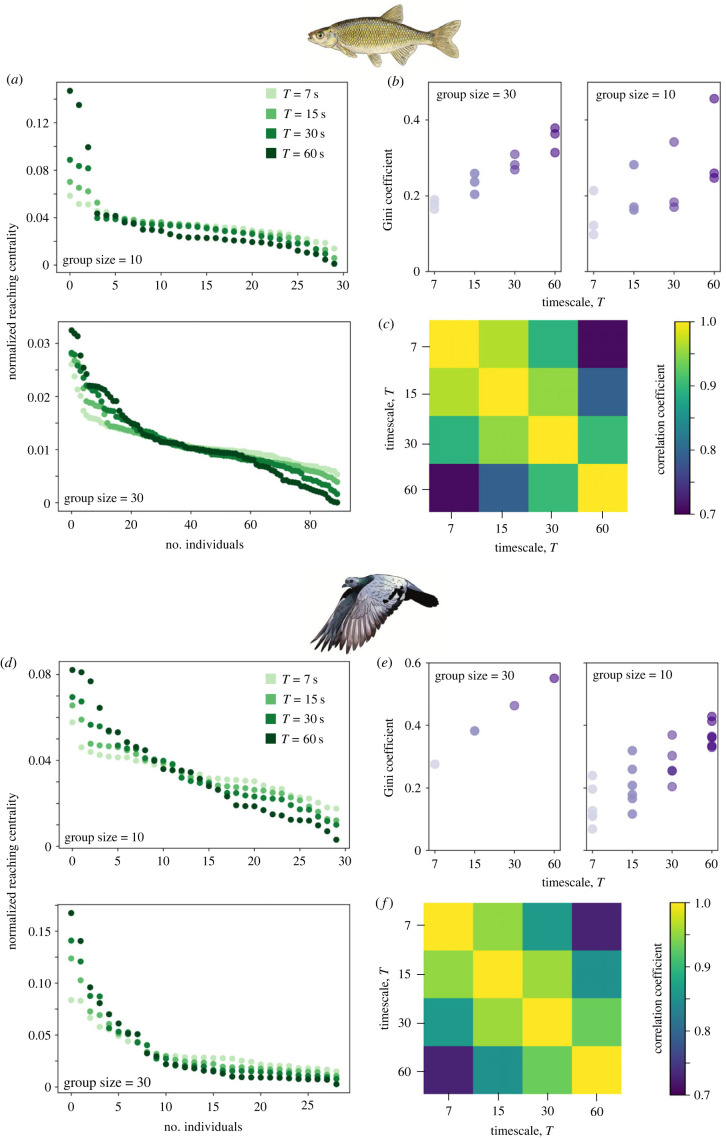
**Distribution of social influence within the group.** Distribution of individual influence scores defined as normalized reaching centralities at four different timescales *T* ∈ {7, 15, 30, 60 s} for groups of 10 and 30 golden shiner fish (*a–c*) and homing pigeons (*d–f*). (*a,d*) At smaller timescales, the distribution of influence is relatively linear within the group, with a smaller slope for the fish compared with the pigeons. However, at larger timescales, between-individual differences in influence add up to result in larger and more consistent inter-individual differences. (*b,e*) This is evidenced by the increase in the Gini coefficient with the timescale of analysis. Finally, (*c,f*) we also calculate the Pearson's correlation coefficient between individual influence scores (*R_k_*) across the different timescales of analysis. While influence scores show strong correlations across the different timescales, a drop in the correlation is seen as the magnitude of the difference between timescales increases.

At the individual level, we also found differences in the distribution of influence across the different timescales, with individual influence scores being more similar for shorter timescales (figure 3*c,f*). For both species, at short timescales the distribution of influence across group members was relatively linearly descending (figure 3*a*,*b*,*d*,*e*). At longer timescales, between-individual differences aggregate, resulting in a larger asymmetry in the influence distribution across group members (indicated by the increasing Gini coefficients with the analysis timescale in figure 3*b*,*e*).

### (b) Temporal dynamics of social influence

**Figure 4.**
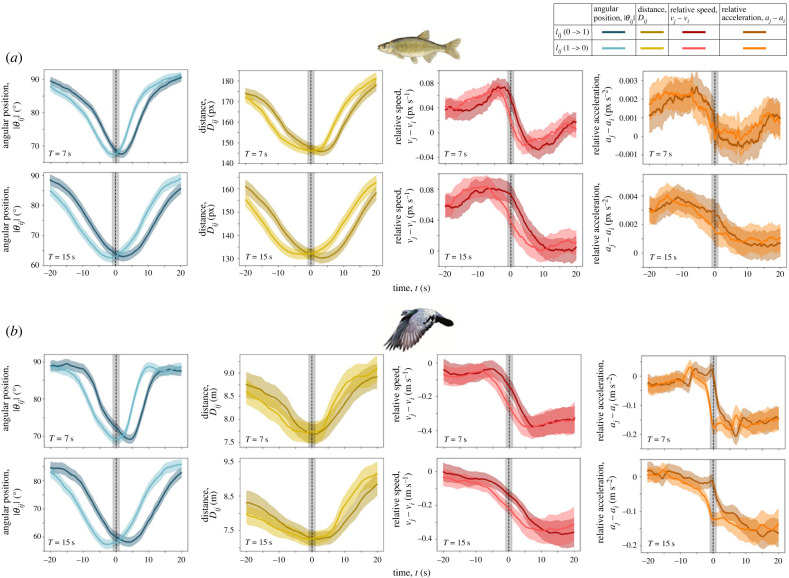
**Temporal dynamics of social influence.** Temporal dynamics of relative position (distance to, and angular position of, the neighbour relative to the focal individual) and relative kinematics (speed and acceleration difference between a focal individual and its neighbour) that accompany a switch in leader–follower dynamics in (*a*) golden shiners and (*b*) homing pigeons. At the black dashed line, the focal individual suddenly decides to follow, or to stop following, its neighbour. The time series are 40 s segments, of which the middle 2 s represent this decision (shown here by the grey-shaded region). The darker lines represent a transition of the focal individual from not following its neighbour to following it (*l_ij_*(0 → 1)), while the lighter lines represent a transition from following to not following (*l_ij_*(1 → 0)). Statistically, these two lines (in each plot) can be perceived as the focal individual giving up followership of one neighbour (represented by the lighter lines) to start following another neighbour (represented by the darker lines). All curves show aggregate data from the entire dataset, and the shaded region shows the 95% confidence intervals around the mean.

Our time series analysis reveals the temporal sequencing of between-individual relationships, before, during and after periods of leadership. Here, we explore the temporal dynamics in angular position, distance, relative speed and relative acceleration among dyads. Here, relative speeds and relative accelerations are measured as the difference in the magnitudes of the velocity and acceleration vectors of the leader and follower, respectively. We consider transitions of the focal individual both from following a neighbour to not following it (*l_ij_*(1 → 0)), and from not following to following (*l_ij_*(0 → 1)). Note that the transition *l_ij_*(1 → 0) could occur because the focal individual *i* switched from following neighbour *j*, to either following a different neighbour *k* or not following any neighbour. Similarly, *l_ij_*(0 → 1) could occur because the focal individual *i* switched from either not following any neighbour or from following some neighbour *k* to following neighbour *j*. Since we simplify our analysis such that the focal individual can only be following one other individual at a given time [39], the dashed line in figure 4 represents a timepoint when, statistically, the focal individual switches the neighbour it is following. For both species, we find that individuals follow close-by neighbours (electronic supplementary material, figure S5) that occupy relatively frontal positions. Changes in relative speed and relative acceleration consistently precede these periods of leadership and individuals exert influence in periods when they are slowing down relative to their neighbours (figure 4). In golden shiners, a switch in leadership, from a focal follower's perspective, coincides with the new leader occupying a closer and a more frontal position compared with the previous leader (figure 4*a*). In homing pigeons, unlike in golden shiners, a switch in the leader is associated only with frontness of the new leader relative to the old one and does not correlate with the difference in closeness of these individuals (figure 4*b*). For both species, kinematic changes (relative speed and relative acceleration) precede leadership, and are therefore reflected in the GLMs (figure 2) only over longer timescales. Individuals are found to occupy leading positions in periods when they slow down relative to the focal follower. While this could be partially driven by kinematic constraints (e.g. individuals must slow down to make large turns), we observe species-specific differences, which suggests that other factors also contribute to this trend. Additionally, in homing pigeons, unlike golden shiners, leader switches are found to be strongly associated with relative accelerations of the focal follower and the two concerned neighbours, i.e. its old and new leader (figure 4).

## 4. Discussion

In this work we present detailed analyses of collective animal movement that demonstrate timescale-dependence of social influence in fish schools and bird flocks. At relatively short timescales (*T* ≤ 15 s)—for both golden shiners and homing pigeons—we find that relative position is the strongest predictor of leadership, and contrary to our expectation, little to no relationship was found between kinematics and leadership. At relatively long timescales (*T* ≥ 30 s), the predictive power of kinematics in relation to leadership increased, but we observed differences between the two study systems. While, in golden shiners, the increase in predictive power of kinematics was modest (and relative position was still the strongest predictor of leadership), in homing pigeons, a reversal in the strength of the different predictors was observed i.e. kinematics became a better predictor of leadership than relative position (figure 2). A time series analysis revealed that this difference in the strength of different predictors at different timescales occurs as a result of the temporal sequencing of behaviour. For both species, changes in leadership were found to coincide with the two individuals coming closer and the leader occupying a more frontal position—seen by the decrease in the values of angular position and distance (figure 4). Changes in kinematics were found to precede leader–follower relationships between dyads, and a decrease in speed was associated with changes in leadership (figure 4). While these changes did not affect individual followership instantaneously, they allowed individuals to occupy influential (frontal and closer) positions, thus increasing their chances of subsequently being followed. This temporal sequencing of kinematic changes preceding leadership is therefore observable only over longer timescales.

Furthermore, our results also suggest that, at short timescales, social influence is relatively linearly distributed across group members, i.e. most or all individuals may exert some influence on group members (figure 3*a*,*b*,*d*,*e*). However over longer timescales, inequality within the group grows (figure 3*b*,*e*), resulting in consistent inter-individual differences in leadership. Our results are in agreement with previous work where context-dependent consistency in leadership has been demonstrated in pigeons [30,36]. Similar results have also been found in golden shiner fish, where it was demonstrated that even when group members did not differ in their knowledge of a food source, individual golden shiner fish differed in their tendency to lead [41] and that only a small proportion of individuals could lead the group to the food source [42].

In general, our work highlights how different interpretations can result from analysing behavioural data at different timescales and demonstrates that analyses at multiple temporal scales are essential for a holistic understanding of the mechanistic and functional underpinnings of collective animal behaviour. We acknowledge that several other approaches have previously been used to quantify social influence—from attraction and alignment force-based approaches [32,43–45] to Bayesian integration [46–48], information theory [49,50] and deep neural network [51] approaches—each with its own set of advantages and limitations. Here, we use the time-lagged directional correlation technique which is agnostic to the exact interaction rules that individuals adopt and has the advantage of directly using movement directions obtained from the data. Our technique not only captures the timescale-dependence of social influence, but also reveals the specificities of leadership that are associated with the different species that we consider. Comparing the two time series considered (*l_ij_*(1 → 0) and *l_ij_*(0 → 1)) for each of the predictor variables (angular position, distance, speed difference and acceleration difference), we reveal how positional and kinematic properties are associated (statistically) with a change in leader. In golden shiners, changes in leadership coincided with the new leader occupying a closer and more frontal position compared with the previous leader. In homing pigeons, unlike in golden shiners, leader switches are associated only with changes in relative angular position of the new leader compared with the previous one, and not with distance—this is seen by the strong overlap between the light yellow and dark yellow lines in figure 4*b* and may result from weak distance-dependence in attraction-like interactions that have been previously described in pigeons [21] and other birds [10]. However, in homing pigeons, a difference in relative acceleration between the old and the new leader also appears to influence leader switches (cf. overlap between the light orange and dark orange lines in figure 4*a*,*b*).

The between-species differences that we observed could partially arise from differences in the physical media in which these animals move. Birds are relatively constrained in their flight speeds. In air, an individual's movement speed is coupled with its flapping activity, which in turn affects its flying altitude. Hence, relative position for flying species—in our case, homing pigeons—is strongly linked to their preferred flight speeds. Fish, however, are able to use their swim bladder to engage in stop-and-go movement, thus regulating their position within the group and proximity to neighbours with higher precision.

Differential leadership has also been considered in other fields. In business and management theory, three levels of leadership are often referred to: bottom-level supervisors, middle-managers, and top-level leaders [52,53]. These different leaders all lead and direct tasks, but the scope and timescale of their leadership differs. While supervisors may make plans for weeks up to months, middle-managers consider longer time periods in their planning (e.g. 1–2 years), and the top-level leaders such as the CEO and board of directors of a company set the strategic direction of the company, which may span multiple years or even decades [53]. Leadership can be analysed across these multiple levels [54], and in addition may serve functions in different areas, such as transformational leadership, ethical leadership and empowering leadership [55]. Similarly, leadership can be considered in different domains. While here we considered movement decisions, other domains of leadership can be regarding resource acquisition, social learning (e.g. others copy the leader's strategy) and mediating within-group conflict or between-group interactions [56]. These examples underscore the importance of considering the timescale and context to interpret leadership in animal groups.

Leadership at short versus long timescales can represent ‘different types’ of leadership. While leaders at short temporal scales may lead particular turns or changes in direction, leaders at longer timescales may set the more general direction of the group. For example, a short-timescale leader may lead the group to avoid an obstacle, but a long-timescale leader may keep the group in the correct direction to return to the home nest or another goal location. While here we only consider positional and kinematic variables as drivers of leadership, there may be several mechanisms that operate at different timescales. The ability to take long-time recordings, which is currently facilitated by new advances in technology, will allow future work to analyse and compare leadership at different timescales. Combining these advances in technology with new analytical techniques and manipulative experiments may help reveal the mechanisms that drive differences in social influence across timescales. For example, applying the methods developed here to mixed groups composed of satiated and starved individuals may reveal the timescale at which hunger affects social influence, and perhaps the kinematic properties via which this influence is manifested. While previous work has primarily aggregated and used data in a timescale-agnostic manner, our results show the importance of considering different timescales and the possible mechanisms that may drive behaviour at each of these timescales.

## Data Availability

All data to support the findings of this study are available with other published papers. Code availability: all analyses were run using custom code written in CUDA and Python and are available in the GitHub repository (https://doi.org/10.5281/zenodo.7113335). The data are also provided in the electronic supplementary material.
